# Pentacam® Corneal Tomography for Screening of Refractive Surgery Candidates: A Review of the Literature, Part I

**Published:** 2019

**Authors:** Mahsaw N. Motlagh, Majid Moshirfar, Michael S. Murri, David F. Skanchy, Hamed Momeni-Moghaddam, Yasmyne C. Ronquillo, Phillip C. Hoopes

**Affiliations:** 1 Department of Ophthalmology, University of Arizona College of Medicine, Tucson, AZ, USA; 2 Department of Ophthalmology and Visual Sciences, John A. Moran Eye Center, School of Medicine, University of Utah, Salt Lake City, UT, USA; 3 HDR Research Center, Hoopes Vision, Draper, UT, USA; 4 Department of Ophthalmology and Visual Sciences, W.K. Kellogg Eye Center, Medical School, University of Michigan, Ann Arbor, MI, USA; 5 Department of Optometry, School of Paramedical Sciences, Mashhad University of Medical Sciences, Mashhad, Iran

**Keywords:** Cornea, Tomography, Refractive Surgery, Pentacam, Keratoconus

## Abstract

Corneal tomography and Scheimpflug imaging are frequently used to analyze the corneal surface, especially in the field of cataract and refractive surgery. The Pentacam system is one of the most commonly used commercially available systems for this purpose. Through a rotating Scheimpflug camera, the system is capable of creating a three-dimensional map of the cornea. These advances in tomography have simultaneously enhanced the ability of clinicians to screen surgical candidates and detect subtle corneal changes in diseases such as keratoconus. However, there remains a need to enhance diagnosis in order to recognize mild and early forms of corneal ectasia. As iatrogenic ectasia and keratoconus are dreaded complications of refractive surgery, it is imperative to screen patients appropriately prior to surgery. The Pentacam is one of many systems utilized in the screening process, but the literature has not identified specific protocol nor parameters that are capable of carrying out this process appropriately. Post-operative keratoconus continues to occur despite the advances in technology seen in corneal imaging. Therefore, clear indices for screening are required in order to diagnose early forms of keratoconus and other corneal diseases that may exclude the seemingly asymptomatic patient from undergoing refractive surgery. This article aims to summarize the indices available on the Pentacam system and to identify the most accurate parameters for screening of the refractive surgery candidate.

## INTRODUCTION

**History of Corneal Tomography**

**Figure 1:**
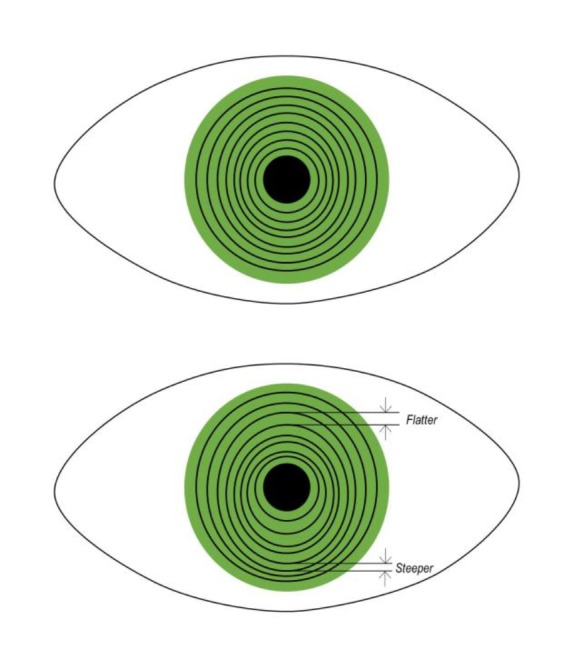
Example of normal (superior) and abnormal (inferior) Placido disk topography. Distortion of the Placido targets is useful in determining topographic changes seen in keratoconus such as inferior steepening

Advancements in corneal and anterior segment imaging have revolutionized ophthalmology over the past twenty-five years allowing for earlier detection of ectasia, particularly in refractive surgery candidates, including those planning for laser assisted in situ-keratomileusis (LASIK). While these advances have proven beneficial in the realm of clinical practice, there is still controversy and disagreement surrounding the refractive indices that should be used in patient evaluation [1-4]. Historically, Placido disk-based corneal topography was an early index introduced in the 1980s that led the way for the evolution of subsequent technological advances [5]. Placido targets refer to a series of illuminated mires that are projected onto the anterior cornea [6]. Disk-based corneal topography captures the Placido targets and calculates corneal curvature based on the size and distortion of the mires (Fig. 1). Klyce and colleagues developed the first indices from computerized analysis of the corneal surface [5, 7-9]. Soon after, Rabinowitz and Rasheed established the Keratometry, Inferior-Superior and Astigmatism (KISA) index expressed in percent for screening patients undergoing refractive surgery [10, 11]. Despite these exciting advances, there were still shortcomings in the measurement of the posterior surface of the cornea and providing a pachymetry map [12]. Three-dimensional tomographic reconstruction of the cornea made possible the determination of the posterior corneal surface through rotational Scheimpflug imaging, optical coherence tomography, and pachymetric mapping [12].

In this review, we focus on the Pentacam Comprehensive Eye Scanner (Oculus Optikgeraete GmbH; Wetzlar, Germany) and its indices for screening patients undergoing cornea refractive procedures such as LASIK and PRK. It employs a rotating Scheimpflug camera that measures elevation points and computes a three-dimensional corneal map through utilization of the Scheimpflug principle [13]. There are other devices based on the Scheimpflug principle, such as TMS-5 (TMS-5 (Tomey Corp., Nagoya, Japan), Sirius (CSO, Costruzione Strumenti Oftalmici, Florence, Italy), and Galilei (Ziemer, Port, Switzerland); however, Pentacam is one of the most commonly used corneal tomographic technologies in clinical practice. This technique allows for three imaginary perpendicular planes (lens, image, and subject) instead of the two in a traditional or normal camera; as a result of the three planes there is an extension of the depth of focus that provides for sharp resolution of the image that coincides with the rotating camera [14]. In a maximum of two seconds, the Oculus Pentacam generates a three-dimensional (3D) model of the anterior segment from as many as 25,000 elevation points and from 138,000 elevations points in the Pentacam HR. Data derived from this device includes top/tomography, pachymetry, chamber angle, chamber volume, chamber height, lens densitometry and many other ocular indices. The use of elevation mapping as its primary data source makes the Pentacam a unique device compared to other modalities [15].

**Function of Corneal Tomography**

Placido-disk based corneal topography quantifies the anterior corneal surface and provides anterior surface curvature data [9, 13, 16]. In contrast, rotating Scheimpflug tomography provides a three-dimensional reconstruction of the entire cornea, including the anterior and posterior surfaces [14]. The Pentacam system is commonly used in clinical practice for its myriad functions, including evaluation of cataracts, glaucoma screening, advanced calculations for the power of intraocular lenses, guiding deep anterior lamellar keratoplasty, and imaging of post-LASIK or photorefractive keratectomy (PRK) eyes. However, the purpose of this article is to focus on its clinical application and utility for screening patients for subtle corneal ectasia. Often, ectatic changes are found prior to loss of visual acuity. The detection of early ectasia provides patients the option to start treatment such as collagen-cross linking that may slow or halt progression of the disease. Ideally, corneal tomography is able to characterize the level of susceptibility each patient has for the development of ectasia [17]. In measuring corneal tomography, the Pentacam system has maintained excellent repeatability and reproducibility in multiple studies [18-24]. Although some studies suggest that Pentacam reliability slightly decreases towards the periphery [25], Pentacam measurements are still far superior to previous Placido-disk analysis and are adequate in the proper diagnosis of peripheral disease such as pellucid marginal degeneration [26, 27]. In a study conducted by McAlinden et al., Pentacam’s precision was the lowest for measuring axes, pupil center pachymetry, front tangential and axial maps, and refractive power maps [22, 28]. When compared to other tomographic devices, such as Galilei and Orbscan II, Pentacam has excellent intra-device precision but inconsistent inter-device measurement repeatability [23, 29, 30]. Ultimately, Pentacam data should always be used in combination with clinical judgment in screening patients.

**Refractive Surgery Screening and Keratoconus Definitions**

Central to screening refractive surgery candidates is the risk of postoperative corneal ectasia, often as a result of keratoconus (KC). Current and past literature is devoid of a consensus definition of KC, specifically in relation to its staging. Traditionally, the diagnosis and grading of KC has been based on the Amsler-Krumeich Classification. This classification system delineates four stages characterized by clinical appearance, central keratometry readings, refraction (myopia and astigmatism), and central corneal thickness [31, 32]. Despite corneal tomography advancements, the classification of KC and keratectasia has remained rooted in this system that was created over fifty years ago.

**Figure 2:**
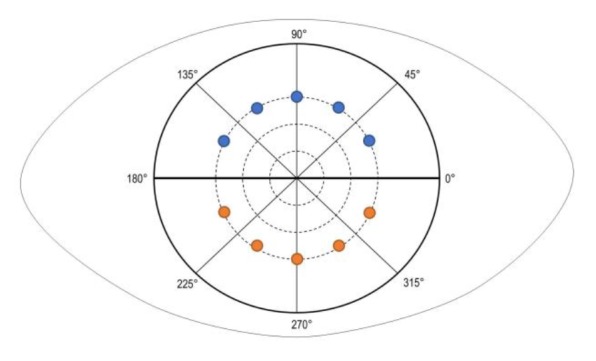
The inferior-superior (I-S) index is calculated as the difference between inferior and superior average dioptric (D) values. The average of five superior points (in blue) above the horizontal meridian are compared to the average of five inferior (in orange) points. The points are spaced in 30-degree intervals and are approximately 3.0-mm from the corneal vertex. Any value >1.4 is suggestive of keratoconus [11, 44].

**Figure 3:**
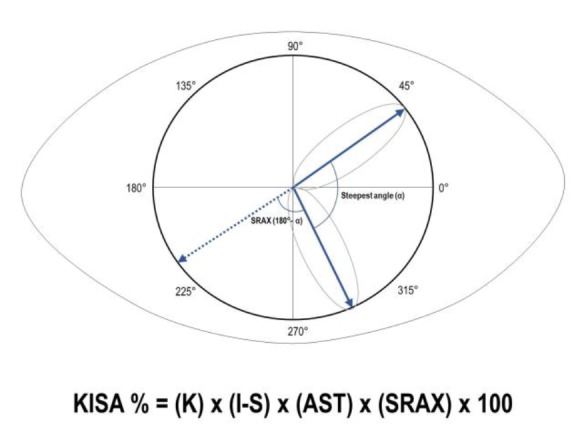
Calculation of the skewed radial axis index, which corresponds to the angle (α) formed between the two steepest semimeridians above and below the horizontal meridian. The SRAX is equal to 180 minus the smaller of the two angles formed by the radii of the semimeridians. The below algorithm for calculating the composite KISA% index. Abbreviations: K: keratometry value; I-S: inferior-superior asymmetry index; AST: degree of regular astigmatism; SRAX: skewed radial axis index

In 2015, the four leading international corneal societies put forth the Global Consensus on Keratoconus and Ectatic Diseases [33] in which they acknowledged that the Amsler-Krumeich classification failed to keep up with technological advances in corneal tomography. However, the Consensus did not define a clinically adequate classification system for KC [33]. Many other studies have created classification systems, including the Cone Location and Magnitude Index (CLMI) [34], Collaborative and Longitudinal Evaluation of Keratoconus (CLEK) Criteria [35], the KISA% index [11], the Keratoconus Severity Score (KSS) [36], the Keratoconus Prediction Index (KPI) and Keratoconus Index (KCI) by Klyce/Maeda [7, 37, 38], the modified Rabinowitz-McDonell criteria [39], and the Ectasia Risk Score System (ERSS) [40]. Fig. 2 provides a graphic representation of the I-S index, which was one of the first local indices used for screening surgical candidates. The I-S index is calculated from local topographic data above and below the horizontal meridian [41]. In Fig. 3, we diagram the KISA% index, which incorporates the I-S index and was one of the first composite indices that quantify topographic features of KC [11]. Piñero and colleagues summarized the topographic patterns of KC as focal steepening located in a zone of protrusion surrounded by concentrically decreasing power zones, focal areas with dioptric (D) values ≥ 47.0 D, inferior-superior (I-S) asymmetry measured to be ≥ 1.4 D, or angling of the hemimeridians in an asymmetric or brokwen bowtie pattern with a skewing of the steepest radial axis (SRAX) [42]. Borderline or form-fruste variants of KC are characterized by the aformentioned abnormal topographic patterns in the absence of slit-lamp or visual acuity changes typical of KC [43].

**Figure 4:**
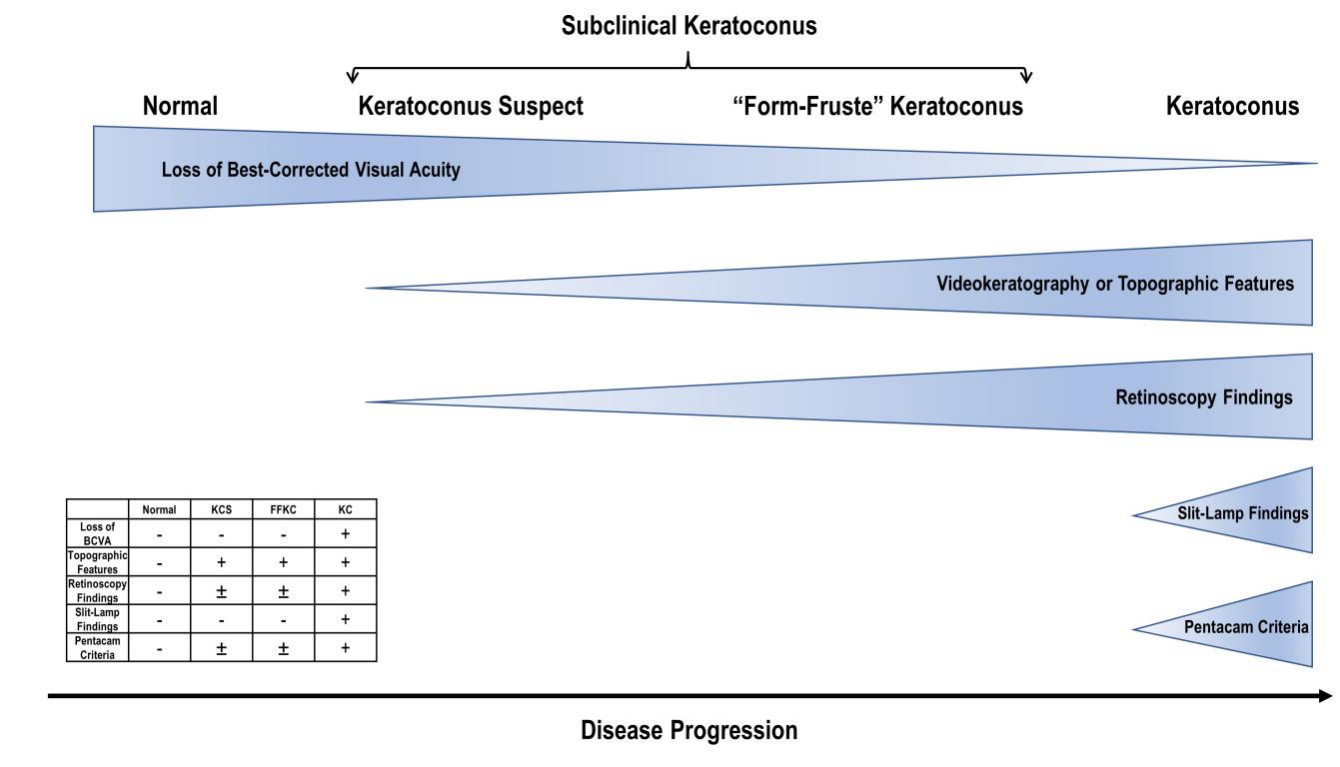
Proposed ectasia spectrum with corresponding clinical characteristics table

Abbreviations: BCVA: best-corrected visual acuity; FFKC: form-fruste keratoconus; KC: keratoconus; KCS: keratoconus suspect.

To this day, there is no universal agreement on whether to classify ectasia based on morphological or topographical patterns, and beyond this, how to categorize subclinical cases. Some have suggested that subclinical KC represents early KC that is only detectable by diagnostic examinations or imaging techniques, and suspect KC describes suspicious topographic features in the absence of clinical signs or diminished visual acuity [45]. From a clinical standpoint, discerning a frank KC is straightforward; but, when it comes to subclinical, form-fruste, suspect, or borderline cases, there is widespread ambiguity [45-49]. The implications of tomographic indices are of the highest value and impact in these cases before the KC is frankly manifest. This patient population is at higher risk of ectasia if subjected to corneal refractive surgery, and also may benefit from stabilization treatment such as corneal crosslinking. While the criteria to define KC suspect, form-fruste KC (FFKC), and subclinical KC are not uniformly established, based on our review these three terms all describe: (1) a topographically normal eye that has frank KC in the fellow eye, or (2) subtle topographic changes without clinical signs of KC or a change in visual acuity [12, 33, 46, 47]. Because the current nomenclature is ambiquous, and to avoid the confusion of these overlapping terms, we propose that the application of corneal tomography is best suited to an ectasia spectrum. For the purposes of screening, the categories of subclinical, form-fruste, suspect, or borderline KC include all patients that the clinician would want to turn away from surgery due to the high post-operative risk for worsening ectasia. Based on our review, a schematic representation of the ectasia spectrum and groups of interest relative to Pentacam screening can be found in Fig. 4. While the purpose of this review article is not to create a universal classification system, it will identify Pentacam derived screening criteria that will advance detection of asymptomatic ectasia. Although many terms such as suspect, borderline, form-fruste, and subclinical exist to describe patients with asymptomatic ectasia, for the purpose of this article any KC that is not symptomatic and not clinically advanced will be referred to as pre-keratoconus.

## METHODS

A literature review was performed using various databases including PubMed, Mendeley, Ovid, Elsevier, and Science Direct. The PubMed primary search term included “Pentacam”, which was connected to descriptors such as “LASIK”, “progression parameters”, “screening”, comparison”, “Scheimpflug”, “tomography”, “keratoconus”, “subclinical keratoconus”, “evaluation”, and others. Peer-reviewed and scholarly resources including original scientific articles as well as review articles were included. Articles were screened for relevance and significance based on their abstracts. Those that were identified as appropriate for this review were included. Additional searches were made to find relevant literature through Mendeley, Ovid, Elsevier, and ScienceDirect. Publications between 1900 and 2019 were included in this review. All articles that were deemed relevant to this topic were included in this review.

Parameters with area under the curve (AUC) > 0.900 were deemed suitable for screening of KC, while parameters with AUC > 0.800 were selected for screening of pre-keratoconus. AUC was selected as the primary inclusion criteria as it inherently evaluates the diagnostic accuracy of a screening parameter. The selected parameters that met these requirements were then incorporated in creating proposed cut-off thresholds. Indices that met these criteria in at least two studies were then averaged based on the cut-off value proposed by the individual study. The highlighted parameters in Tables 1-4 indicate the selected averaged cut-off values.

**Table 1:** Studies Evaluating Topometric and Topographic Pentacam Parameters in Detecting Clinical Keratoconus

Study	Cut-off Value	Sensitivity	Specificity	AUC
CKI				
Orucoglu et al [50]	1.015	0.727	0.982	0.824
Chan et al [51]	1.02	0.670	0.950	0.840
Shetty et al [52]	1.03*	0.792	0.686	0.900
Hashemi et al [53]	1.014	0.652	0.970	0.714
Uçakhan et al [54]	1.015	0.864	0.857	0.877
KI				
Orucoglu et al [50]	1.055	0.910	0.982	0.970
Chan et al [51]	1.050	0.870	0.910	0.854
Huseynli et al [55]	1.04*	0.933	0.979	0.994
Shetty et al [52]	1.07	1.000	0.657	0.954
Uçakhan et al [54]	1.125	0.864	0.857	0.907
IHA				
Kovács et al [56]	-	0.850	0.870	0.900
Orucoglu et al [50]	8.65	0.757	0.886	0.883
Chan et al [51]	12.15*	0.730	0.910	0.913
Shetty et al [52]	21	0.708	0.800	0.892
Hashemi et al [53]	9.5	0.707	0.929	0.872
Luz et al [57]	10.1	0.723	0.956	0.890
Uçakhan et al [54]	11.6	0.841	0.794	0.852
IHD				
Kovács et al [56]	-	0.950	0.980	0.970
Orucoglu et al [50]	0.0175	0.900	0.890	0.951
Chan et al [51]	0.020	0.930	0.910	0.975
Lopes et al [58]	0.0205*	0.995	0.988	1.000
Huseynli et al [55]	0.013	0.967	0.969	0.979
Haddad et al [59]	0.015	0.971	0.988	-
Shetty et al [52]	0.016	1.000	0.457	0.968
Hashemi et al [53]	0.012	0.900	0.906	0.949
Luz et al [57]	0.021	0.893	0.985	0.974
Uçakhan et al [54]	0.0335	0.909	0.794	0.886
ISV				
Orucoglu et al [50]	31.5	0.878	0.962	0.954
Chan et al [51]	24.0	0.930	0.910	0.990
Lopes et al [58]	32.5*	0.945	0.987	0.995
Shetty et al [52]	41	1.000	0.700	0.972
Hashemi et al [53]	33	0.803	0.946	0.945
Luz et al [57]	35	0.904	0.980	0.977
Uçakhan et al [54]	59	0.841	0.905	0.924
IVA				
Orucoglu et al [50]	0.255	0.913	0.964	0.963
Chan et al [51]	0.160	0.930	0.910	0.992
Huseynli et al [55]	0.240*	0.978	0.958	0.996
Shetty et al [52]	0.32	1.000	0.700	0.955
Hashemi et al [53]	0.20	0.875	0.963	0.952
Luz et al [57]	0.32	0.876	0.976	0.958
Uçakhan et al [54]	0.455	0.909	0.841	0.903
Rmin				
Orucoglu et al [50]	7.085	0.968	0.807	0.929
Chan et al [51]	7.320*	0.930	0.910	0.984
Shetty et al [52]	6.71	0.917	0.771	0.771
Hashemi et al [53]	7.21	0.919	0.833	0.932
Uçakhan et al [54]	6.545	0.952	0.636	0.943
PE				
Kovács et al [56]	-	0.960	0.950	0.980
Xu et al [60]	30.165	0.940	0.880	0.970
Lopes et al [58]	16.5	0.945	0.979	0.988
Mihaltz et al [61]	15.5	0.951	0.943	0.970
Huseynli et al [55]	11.0	1.000	0.994	0.999
Haddad et al [59]	14.0	0.962	0.989	-
Ambrósio et al [14]	12.0	0.963	0.988	0.991
Ambrósio et al [14]	8.0	0.950	0.991	0.994
Jafarinasab et al [62]	35.0	0.939	0.886	0.977
Uçakhan et al [54]	26.5	0.977	0.810	0.926
De Sanctis et al [63]	35.0	0.973	0.969	0.990
Muftuoglu et al [64]	15.0*	0.980	1.000	0.999
Kamiya et al [65]	14.0	0.921	0.952	0.898

Abbreviations: AUC: area under the curve; CKI: central keratoconus index; KI: keratoconus index; IHA: index of height asymmetry; IHD: index of height decentration; ISV: index of surface variance; IVA: index of vertical asymmetry; Rmin: minimum radius of curvature; PE: posterior elevation. *denotes cut-off point with best area under the curve if more than one study evaluated index accuracy. Highlighted indices represent the parameters that were included in the final evaluation of proposed thresholds.

**Table 2:** Studies Evaluating Topometric and Topographic Pentacam Parameters in Detecting Pre-Keratoconus

Study	Cut-off Value	Sensitivity	Specificity	AUC
CKI				
Shetty et al [52]	1.03	0.0027	0.977	0.576
Uçakhan et al [54]	1.005*	0.773	0.413	0.683
KI				
Huseynli et al [55]	1.03*	0.867	0.875	0.810
Shetty et al [52]	1.07	1.000	0.000	0.631
Uçakhan et al [54]	1.05	0.864	0.635	0.794
IHA				
Kovács et al [56]	-	0.670	0.500	0.610
Shetty et al [52]	19	1.000	1.000	0.611
Uçakhan et al [54]	7.10*	0.682	0.619	0.682
IHD				
Kovács et al [56]	-	0.800	0.750	0.880
Bae et al [66]	0.008	0.714	0.853	0.748
Huseynli et al [55]	0.008*	0.823	0.650	0.782
Shetty et al [52]	0.014	0.432	0.674	0.627
Uçakhan et al [54]	0.0135	0.750	0.603	0.703
ISV				
Hashemi et al [67]	22*	0.745	0.618	0.800
Shetty et al [52]	37	1.000	0.962	0.739
Uçakhan et al [54]	24.5	0.864	0.667	0.795
IVA				
Bae et al [66]	0.160	0.714	0.618	0.733
Huseynli et al [55]	0.150	0.921	0.525	0.844
Hashemi et al [67]	0.140*	0.823	0.732	0.860
Shetty et al [52]	0.28	0.108	0.953	0.609
Uçakhan et al [54]	0.195	0.864	0.619	0.768
Rmin				
Uçakhan et al [54]	7.275*	0.698	0.614	0.697
PE				
Kovács et al [56]	-	0.730	0.710	0.750
Xu et al [60]	12.335	0.930	0.670	0.856
Bae et al [66]	11.10	0.571	0.882	0.735
Huseynli et al [55]	8.0	0.955	0.763	0.870
Ambrósio et al [14]	5.0	0.745	0.749	0.825
Ambrósio et al [14]	1.0	0.809	0.725	0.849
Jafarinasab et al [62]	14.0	0.927	0.090	0.698
Uçakhan et al [54]	20.5	0.818	0.667	0.789
De Sanctis et al [63]	29.0*	0.680	0.908	0.930
Du et al [68]	7.5	0.707	0.938	0.882
Muftuoglu et al [64]	11.0	0.530	0.900	0.709

Abbreviations: AUC: area under the curve; CKI: central keratoconus index; KI: keratoconus index; IHA: index of height asymmetry; IHD: index of height decentration; ISV: index of surface variance; IVA: index of vertical asymmetry; Rmin: minimum radius of curvature; PE: posterior elevation. *denotes cut-off point with best area under the curve. Highlighted indices represent the parameters that were included in the final evaluation of proposed thresholds.

**Table 3:** Studies Evaluating Pachymetric Pentacam Parameters in Detecting Clinical Keratoconus

Study	**Cut-off Value**	**Sensitivity**	**Specificity**	**AUC**
ART-Min				
Muftuoglu et al [69]	607	0.950	0.920	0.968
Muftuoglu et al [64]	604*	0.910	0.990	0.973
ART-Max				
Kovács et al [56]	-	0.960	0.950	0.890
Orucoglu et al [50]	311	0.966	0.907	0.961
Chan et al [51]	342	0.930	0.910	0.973
Sedaghat et al [70]	312	0.986	0.993	0.986
Lopes et al [58]	317.5	0.978	0.983	0.995
Haddad et al [59]	344	0.958	0.985	-
Shetty et al [52]	390	1.000	0.557	0.958
Muftuoglu et al [69]	301	0.990	0.950	0.991
Muftuoglu et al [64]	313	0.930	1.000	0.985
Lim et al [71]	339	-	-	0.865
Ruiseñor Vázquez et al [72]	349	0.905	0.909	0.950
Wahba et al [73]	412	0.973	0.932	0.987
Ambrósio et al [74]	339	1.000	0.956	0.983
Ambrósio et al [14]	386*	0.992	0.973	0.999
Luz et al [57]	496	0.989	0.990	0.997
ART-Avg				
Chan et al [51]	431.50	0.870	0.910	0.962
Lopes et al [58]	435.5	0.984	0.968	0.992
Haddad et al [59]	473	0.979	0.974	-
Muftuoglu et al [69]	407	0.980	0.940	0.989
Muftuoglu et al [64]	392	0.930	0.990	0.963
Lim et al [71]	424	-	-	0.832
Ruiseñor Vázquez et al [72]	459	0.889	0.855	0.920
Wahba et al [73]	496	0.945	0.942	0.976
Ambrósio et al [74]	424	0.955	0.965	0.987
Ambrósio et al [14]	474*	0.996	0.982	0.999
Luz et al [57]	474	0.983	0.990	0.997
BAD_D				
Orucoglu et al [50]	2.615	0.932	0.990	0.972
Chan et al [51]	2.00	1.000	0.910	0.994
Sedaghat et al [70]	2.31*	1.000	1.000	1.000
Ferreira-Mendes et al [75]	0.575	0.914	0.955	0.981
Lopes et al [58]	2.33	0.989	0.987	0.999
Huseynli et al [55]	1.83	1.000	0.959	0.993
Haddad et al [59]	2.32	0.982	1.000	-
Hashemi et al [67]	2.38	0.967	0.948	0.990
Shetty et al [52]	2.60	1.000	0.614	0.972
Ambrósio et al [74]	2.11*	0.996	1.000	1.000
Luz et al [57]	1.34*	1.000	0.985	1.000
Muftuoglu et al [64]	2.10*	1.000	1.000	1.000
CCT				
Sedaghat et al [70]	519	0.931	0.912	0.978
Hosseini et al [76]	503.3	0.921	.0887	0.830
Lopes et al [58]	509.5	0.780	0.895	0.920
Demir et al [77]	484.5*	0.968	9.933	0.993
Reddy et al [78]	534	0.710	0.920	0.860
Toprak et al [79]	519	0.891	0.908	0.946
Muftuoglu et al [69]	494	0.820	0.710	0.811
Uçakhan et al [54]	502.5	0.905	0.636	0.832
Muftuoglu et al [64]	511	0.870	0.710	0.832
Ahmadi Hosseni et al [80]	503	0.921	0.887	0.830
Dienes et al [81]	513	0.910	0.930	0.920
Shetty et al [82]	516	0.920	0.841	0.930
Labiris et al [83]	529	0.818	0.871	0.900
Ambrósio et al [74]	529	0.955	0.730	0.909
PPI-Min				
Orucoglu et al [50]	0.925	0.854	0.972	0.935
Ambrósio et al [74]	0.790	0.932	0.858	0.939
Muftuoglu et al [69]	0.930	0.920	0.900	0.957
Uçakhan et al [54]	0.850	0.909	0.825	0.928
Muftuoglu et al [64]	0.840*	0.890	0.960	0.960
PPI-Max				
Kovács et al [56]	-	0.960	0.960	0.970
Orucoglu et al [50]	1.675	0.888	0.978	0.974
Sedaghat et al [70]	1.6	0.986	0.978	0.998
Huseynli et al [55]	1.54	0.978	0.938	0.975
Ambrósio et al [74]	1.44	1.000	0.938	0.977
Muftuoglu et al [69]	1.69	0.950	0.920	0.980
Wahba et al [73]	1.40	0.918	0.981	0.987
Luz et al [57]	1.42*	0.955	0.955	0.995
Uçakhan et al [54]	1.45	0.977	0.730	0.934
Muftuoglu et al [64]	1.56	0.930	1.000	0.966
PPI-Avg				
Orucoglu et al [50]	1.185	0.914	0.951	0.955
Sedaghat et al [70]	1.20*	0.979	0.993	0.998
Huseynli et al [55]	1.21	0.978	0.907	0.960
Shetty et al [52]	1.06	0.958	0.471	0.962
Ambrósio et al [74]	1.06	0.977	0.985	0.980
Muftuoglu et al [69]	1.29	0.950	0.910	0.976
Wahba et al [73]	1.10	0.877	0.981	0.978
Luz et al [57]	1.05	0.977	0.985	0.995
Uçakhan et al [54]	1.35	0.955	0.841	0.943
Muftuoglu et al [64]	1.25	0.930	0.990	0.955
PRFI				
Lopes et al [58]	0.478*	1.000	0.997	0.999
Haddad et al [59]	0.15	0.943	0.998	-
TCT				
Kovács et al [56]	-	0.910	0.870	0.980
Xu et al [60]	479.50	0.830	0.780	0.862
Orucoglu et al [50]	506.50	0.890	0.832	0.915
Sedaghat et al [70]	512	0.952	0.927	0.986
Hosseini et al [76]	489.1	0.941	0.891	0.850
Lopes et al [58]	503.5	0.863	0.916	0.955
Haddad et al [59]	514	0.893	0.910	-
Demir et al [77]	453.0*	0.994	0.933	0.994
Montalbán et al [84]	525.9	0.984	0.710	0.950
Reddy et al [78]	500	0.660	0.990	0.840
Toprak et al [79]	513	0.896	0.933	0.956
Muftuoglu et al [69]	489	0.900	0.790	0.897
Uçakhan et al [54]	493.5	0.921	0.737	0.896
Muftuoglu et al [64]	501	0.920	0.680	0.873
Ahmadi Hosseni et al [80]	489.1	0.941	0.891	0.850
Dienes et al [81]	509	0.930	0.890	0.940
Shetty et al [82]	509	0.833	0.841	0.860
Labiris et al [83]	522	0.886	0.859	0.940
Ambrósio et al [74]	504	0.955	0.841	0.955
Hashemi et al [53]	507	0.875	0.963	0.952

Abbreviations: AUC: area under the curve; ART-Min: minimum Ambrósio relational thickness; ART-Max: maximum Ambrósio relational thickness; ART-Avg: average Ambrósio relational thickness; BAD_D: Belin-Ambrósio enhanced ectasia display total deviation value (D); CCT: central corneal thickness; PPI-Min: minimum pachymetric progression index; PPI-Max: maximum pachymetric progression index; PPI-Avg: average pachymetric progression index; PRFI: Pentacam random forest index; TCT: thinnest corneal thickness. *denotes cut-off point with best area under the curve if more than one study evaluated index accuracy. Highlighted indices represent the parameters that were included in the final evaluation of proposed thresholds.

**Table 4:** Studies Evaluating Pachymetric Pentacam Parameters in Detecting Pre-Keratoconus

Study	Cut-off Value	Sensitivity	Specificity	AUC
ART-Min				
Muftuoglu et al [69]	691	0.700	0.610	0.714
Steinberg et al [85]	725	0.370	0.367	0.317
Muftuoglu et al [64]	781*	0.680	0.730	0.739
ART-Max				
Kovács et al [56]	-	0.840	0.540	0.740
Shetty et al [52]	340	0.865	0.698	0.850
Muftuoglu et al [69]	372	0.730	0.630	0.739
Muftuoglu et al [64]	408	0.670	0.710	0.722
Ruiseñor Vázquez et al [72]	349	0.905	0.865	0.930
Ambrósio et al [14]	416*	0.851	0.931	0.959
Steinberg et al [85]	412	0.308	0.306	0.272
ART-Avg				
Muftuoglu et al [69]	487	0.720	0.600	0.722
Muftuoglu et al [64]	485	0.610	0.740	0.693
Ruiseñor Vázquez et al [72]	459	0.889	0.784	0.880
Ambrósio et al [14]	521*	0.915	0.931	0.956
Steinberg et al [85]	522	0.342	0.342	0.305
BAD_D				
Ferreira-Mendes et al [75]	0.325	0.684	0.846	0.839
Huseynli et al [55]	1.59	0.955	0.737	0.904
Hashemi et al [67]	1.54	0.811	0.732	0.860
Shetty et al [52]	1.60	0.838	0.860	0.887
Ruiseñor Vázquez et al [72]	1.61	0.892	0.823	0.930
Ambrósio et al [14]	1.22*	0.936	0.946	0.975
Steinberg et al [85]	1.4	0.658	0.658	0.712
Muftuoglu et al [64]	1.31	0.600	0.900	0.834
CCT				
Cui et al [86]	511.5*	0.737	0.966	0.887
Reddy et al [78]	539	0.610	0.820	0.770
Muftuoglu et al [69]	519	0.580	0.540	0.601
Uçakhan et al [54]	511.5	0.778	0.614	0.767
Muftuoglu et al [64]	527	0.660	0.520	0.617
Du et al [68]	523.5	0.781	0.810	0.852
PPI-Min				
Cui et al[86]	0.95*	0.790	1.000	0.942
Ruiseñor Vázquez et al [72]	0.76	0.730	0.737	0.790
Muftuoglu et al [69]	0.62	0.760	0.640	0.795
Steinberg et al [85]	0.70	0.637	0.653	0.657
Uçakhan et al [54]	0.65	0.864	0.683	0.820
Muftuoglu et al [64]	0.66	0.690	0.700	0.714
PPI-Max				
Kovács et al [56]	-	0.670	0.690	0.790
Cui et al [86]	1.45*	0.895	0.931	0.970
Huseynli et al [55]	1.28	0.966	0.579	0.844
Ruiseñor Vázquez et al [72]	1.41	0.865	0.871	0.920
Muftuoglu et al [69]	1.32	0.780	0.650	0.813
Steinberg et al [85]	1.30	0.664	0.668	0.712
Uçakhan et al [54]	1.55	0.841	0.778	0.840
Muftuoglu et al [64]	1.26	0.640	0.640	0.679
PPI-Avg				
Cui et al [86]	1.05*	0.947	0.897	0.957
Huseynli et al [55]	1.14	0.933	0.474	0.834
Shetty et al [52]	1.06	0.838	0.744	0.883
Ruiseñor Vázquez et al [72]	1.09	0.784	0.828	0.860
Muftuoglu et al [69]	0.98	0.770	0.650	0.806
Steinberg et al [85]	1.0	0.623	0.643	0.669
Uçakhan et al [54]	1.15	0.818	0.778	0.842
Muftuoglu et al [64]	1.15	0.540	0.730	0.629
TCT				
Kovács et al [56]	-	0.640	0.660	0.670
Xu et al [60]	498.835	0.920	0.470	0.695
Cui et al [86]	506.5*	0.842	1.000	0.914
Reddy et al [78]	532	0.700	0.800	0.790
Muftuoglu et al [69]	512	0.640	0.580	0.652
Uçakhan et al [54]	497.5	0.889	0.614	0.805
Muftuoglu et al [64]	515	0.680	0.540	0.639
Steinberg et al [85]	524	0.363	0.357	0.323

Abbreviations: AUC: area under the curve; ART-Min: minimum Ambrósio relational thickness; ART-Max: maximum Ambrósio relational thickness; ART-Avg: average Ambrósio relational thickness; BAD_D: Belin-Ambrósio enhanced ectasia display total deviation value (D); CCT: central corneal thickness; PPI-Min: minimum pachymetric progression index; PPI-Max: maximum pachymetric progression index; PPI-Avg: average pachymetric progression index; TCT: thinnest corneal thickness. *denotes cut-off point with best area under the curve if more than one study evaluated index accuracy. Highlighted indices represent the parameters that were included in the final evaluation of proposed thresholds.

**Indices for Refractive Screening**

**Figure 5:**
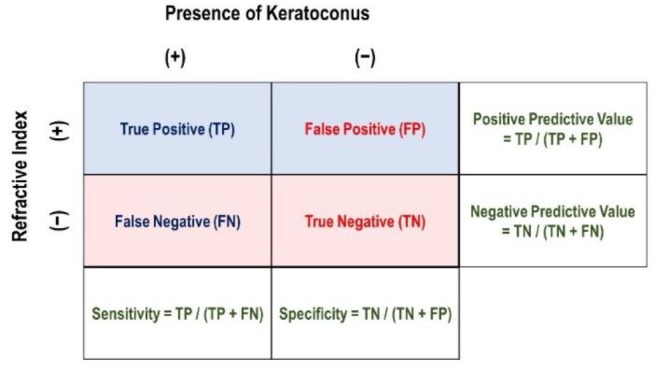
Calculations for sensitivity, specificity, positive predictive value, and negative predictive value based on the presence or absence of keratoconus and refractive index

The Pentacam refractive indices include a combination of tomographic, topometric, and pachymetric parameters. While the Pentacam device is capable of vast data output, our focus is to summarize the best screening indices and compare them to each other in order to provide a quick screening guideline that can be applied in daily clinical practice. Each index described is evaluated based on sensitivity (SN), specificity (SP), and AUC. SN is a criterion that describes the ability of a refractive index to detect a particular disease, in this case KC [41]. As SN increases, the rate of false-negatives decreases; therefore, if SN of a test is 100%, then the false-negative rate is zero. Conversely, SP is a criterion that describes the ability of a refractive index to identify true-negatives. In simpler terms, SP characterizes the proportion of patients without disease who test negative [41]. If the SP of a test is 100%, then the false-positive rate is zero. A highly sensitive test is capable of ruling out disease, while a highly specific test is capable of ruling in disease. Fig. 5 shows the positive and negative predictive value of a test.

**Figure 6:**
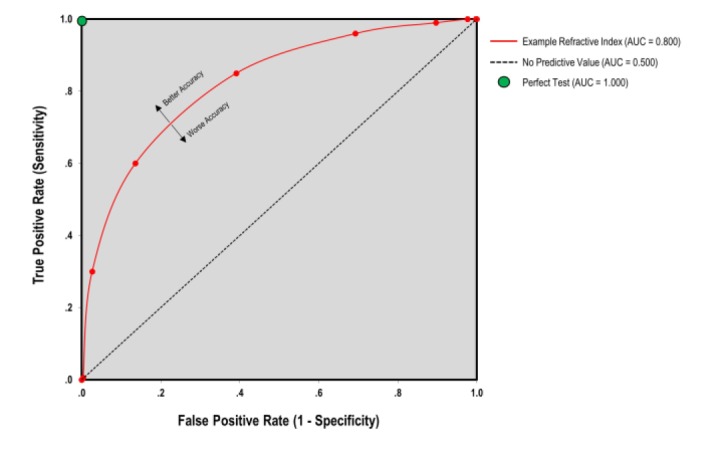
Area under the curve as determined by true-positive rate against the false-positive rate. Diagnostic accuracy is the area under the red line. The dashed line is equal to an area under the curve of 0.500, which is as accurate as a random guess

Studies that evaluate refractive indices rely on AUC to characterize test accuracy. The AUC is simply a measure of how well a parameter can distinguish between two diagnostic groups, which in our case refers either to KC versus normal or pre-keratoconus versus normal (Fig. 6). The diagnostic accuracy of an index based on the AUC is classified as either excellent (> 0.9), good (0.8 to 0.9), fair (0.7 to 0.8), or poor (0.6 to 0.7). An AUC < 0.6 is considered a “fail” and should not be used to distinguish diagnostic groups.

**Figure 7:**
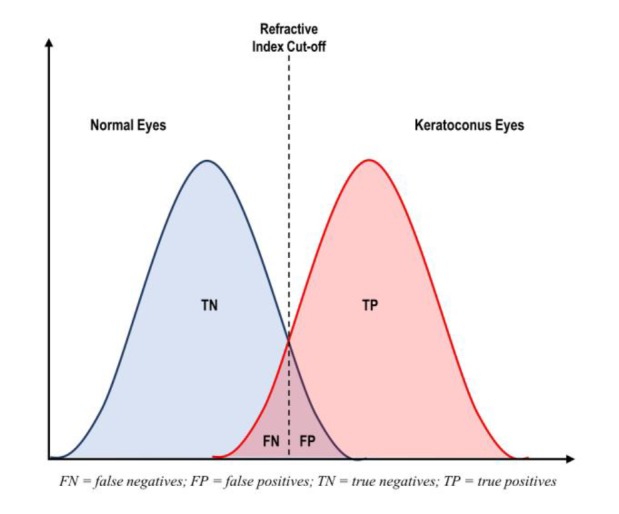
The role of refractive index cut-off as demonstrated by the occurrence of false-positives or false-negatives

The ideal screening test would have 100% SN and SP. However, as the cutoff value for a particular refractive index is increased, there is an expected increase in the false-negative rate. Similarly, if the cutoff value is decreased, then the rate of false-positives is increased. This dynamic relationship between cutoff and predictive values of a test is shown in Fig. 7.

**Table 5:** Abnormality Ranges of Anterior Surface Indices Provided by the Pentacam System

**Parameter**	**Abnormal (Yellow)**	**Pathological (Red)**
**CKI**	≥ 1.03	≥ 1.03
**KI**	> 1.07	> 1.07
**IHA**	≥ 19	> 21
**IHD**	≥ 0.014	> 0.016
**ISV**	≥ 37	≥ 41
**IVA**	≥ 0.28	≥ 0.32
**Rmin**	< 6.71	< 6.71

Abbreviations: CKI: central keratoconus index; KI: keratoconus index; IHA: index of height asymmetry; IHD: index of height decentratio; ISV: index of surface variance; IVA: index of vertical asymmetry; Rmin: minimum radius of curvature.

**Table 6:** The Clinical “Cheat Sheet”: Suggested Cut-off Values for Keratoconus Indices in Screening Clinical and Subclinical Cases

Parameter	Clinical Keratoconus	Subclinical Keratoconus
Tomographic	**Cut-Off Value**	**Cut-Off Value**
**CKI**	**	**
**KI**	1.07	**
**IHA**	10.4	**
**IHD**	0.017	**
**ISV**	36.6	**
**IVA**	0.28	0.15
**Rmin**	7.04	**
**PE**	20.5	10.5
Pachymetric		
**ART-Min**	606	**
**ART-Max**	356	368
**ART-Avg**	444	490
**BAD_D**	2.02	1.31
**CCT**	515	518
**PPI-Min**	0.87	0.80
**PPI-Max**	1.53	1.40
**PPI-Avg**	1.18	1.08
**TCT**	506	502

Abbreviations: CKI: central keratoconus index; KI: keratoconus index; IHA: index of height asymmetry; IHD: index of height decentration; ISV: index of surface variance; IVA: index of vertical asymmetry; Rmin: minimum radius of curvature; PE: posterior elevation; ART-Min: minimum Ambrósio relational thickness; ART-Max: maximum Ambrósio relational thickness; ART-Avg: average Ambrósio relational thickness; BAD_D: Belin-Ambrósio enhanced ectasia display total deviation value (D); CCT: central corneal thickness; PPI-Min: minimum pachymetric progression index; PPI-Max: maximum pachymetric progression index; PPI-Avg: average pachymetric progression index; TCT: thinnest corneal thickness. **denotes insufficient data to conclude a recommendation for screening.

**Table 7:** Belin/Ambrósio Enhanced Ectasia Display Values

Display Value	Description of Parameter
Df	Standard deviation from the mean anterior elevation
Db	Standard deviation from the mean of posterior elevation
Dp	Standard deviation from the mean of average pachymetric progression
Dt	Standard deviation from the mean of thinnest corneal thickness
Dy	Standard deviation from the mean displacement of the thinnest point along the vertical meridian
Da	Standard deviation from the mean of Ambrósio relational thickness
D	Final overall map reading

A side-by-side comparison of the indices studied, along with cut-off values, SN, SP, and AUC for both clinical and pre-keratoconus cases can be found in Tables 1-4. For the reference of our clinicians, the abnormality ranges for the standard Pentacam topographic parameters are summarized in Table 5. Based on our comprehensive review, a simplified version of the KC screening indices along with our recommended cut-off values can be found in Table 6.

**Topometric and Topographic Indices**

Topometric and topographic indices of the Pentacam analyzes and evaluates the surface of the cornea in an objective manner. A variety of automated indices are provided by the Pentacam system. The following subsections detail the indices available through the Pentacam system for screening purposes. These sections provide extensive detail on the background and statistical power of each index, which may be used as a reference. For practical clinical purposes, there are certain caveats: (1) although there are many indices that are validated for detecting KC versus normal eyes, many indices perform poorly or are not validated in the discrimination of pre-keratoconus and (2) there is utility in using more than one single screening index, but also in not using too many. Using several key indices allows the clinician to corroborate findings but with the added risk of losing vital data in overload.

Cognizant of these caveats, we recommend the following indices as the most effective for detection of pre-keratoconus: Belin-Ambrósio Enhanced Ectasia Display Total Deviation Value (BAD_D), Ambrósio relational thickness (ART) values, pachymetric progression indices (PPI), and the index of vertical asymmetry (IVA).

**Anterior Elevation**

With the advent of Scheimpflug imaging, anterior elevation measurement has become readily attainable with great precision. Height data is valuable in directly assessing protrusion, which is important in the diagnosis of KC. We have included this section on anterior elevation for the purpose of completeness, but the overwhelming majority of studies have validated posterior elevation and posterior elevation differences to be superior [30, 61, 62, 65, 73, 87-89]. While anterior elevation is sensitive in detecting frank KC, it is not a reliable parameter in discriminating pre-keratoconus. For this reason, we recommend incorporating anterior elevation data in the context of other diagnostic parameters and focusing on posterior elevation as a superior screening index.

**Central Keratoconus Index**

The central keratoconus index (CKI) is the ratio between the mean radius of curvature values in a peripheral Placido ring divided by a central ring [90]. Slit-scanning devices like Pentacam are capable of directly measuring anterior and posterior elevation. The elevation map determines the height of the cornea relative to a reference shape, which is defined by the radius of curvature that best matches the average corneal curvature [13, 91]. Steeper corneal curvatures translate to smaller radii of curvature. 

As a refractive screening parameter, CKI is highly capable of discriminating clinical KC from normal eyes [51, 53-55, 66]. This includes a recent study by Bae and colleagues that evaluated the subtle morphologic changes of pre-keratoconus comparing the fellow eye of individuals with unilateral KC to normal control groups [66]. The rationale for this approach is inspired by the notion that true unilateral KC is extremely rare, and that the normal fellow eye of an individual with “unilateral” KC is the ideal model for identifying early ectatic changes. In their analysis, CKI, along with nearly all other parameters, successfully delineated between KC and normal eyes. However, it failed to discern between controls and fellow eyes of patients with KC [66]. This is reaffirmed in a more recent study that further demonstrated CKI as a poor parameter in discerning pre-keratoconus [55]. We recommend the use of CKI with caution as a sole parameter in delineating KC from normal. Furthermore, the applicability of CKI to pre-keratoconus cases is limited and requires further validation before relying on it as a parameter for refractive screening. Nevertheless, CKI is a valuable index that can provide a quick reference in identifying frank KC and can also serve to reaffirm clinical suspicion as a cross-reference parameter relative to other indices. 

**Keratoconus Index**

The keratoconus index (KI) is defined as the ratio between mean radius of curvature values in the upper and lower corneal segments [90]. Like CKI, the KI is an efficient diagnostic test to discriminate normal eyes from clinical KC. In a study conducted by Orucoglu and associates, KI was superior to CKI [50]. In a later study, Orucoglu and Toker also demonstrated that KI is an excellent diagnostic indicator with both SN and SP >90% in discrimination analysis of KC [92]. However, this predictive accuracy was not seen in a study by Chan et al, which showed a less robust diagnostic accuracy when comparing KI to other Pentacam parameters [51]. In either study, however, KI was superior to CKI in distinguishing KC. 

Despite the promise of KI in discriminating normal corneas from frank KC, it is limited in its application to pre-keratoconus [55, 66]. We advise caution when using KI for pre-keratoconus given its suboptimal diagnostic accuracy. However, for detection of eyes with KC the literature suggests KI is a reliable parameter that can be used for screening period.

**Index of Height Asymmetry**

The index of height asymmetry (IHA) characterizes the level of elevation symmetry data with respect to the horizontal meridian [42]. As shown in Table 1 IHA is a highly sensitive parameter that has demonstrated excellent diagnostic accuracy for detection of KC [52, 55, 66]. Unfortunately, in the same studies that validate its use for KC, IHA has been identified as a poor parameter for detection of pre-keratoconus (Table 2) [52, 55, 66]. This recurring pattern highlights the crucial shortcoming of each index in identifying the early ectatic changes. IHA is similar to the aforementioned indices in the sense that it should not be used alone in identifying early ectasia. 

**Index of Height Decentration**

The index of height decentration (IHD) is an estimation calculated from a Fourier analysis. This index provides the degree of centration in the vertical direction, calculated on a ring with a radius of 3.0-mm [42]. Overall, IHD echoes the pattern described before [50, 52, 93], but with the added benefit of potentially being able to discriminate pre-keratoconus cases (Table 2). When comparing normal and fellow eyes of individuals with unilateral KC, Bae et al showed that IHD was significantly different and thus useful in identifying topographic changes in unilateral cases [66]. This confirmed in a later study in which IHD maintained very good diagnostic accuracy when comparing bilateral pre-keratoconus to normal controls [55]. 

Despite the promising results for IHD in the aforementioned studies, there are inconsistencies in the literature in regard to its application for pre-keratoconus cases. Some studies have concluded that IHD lacks the accuracy required for a reliable clinical test [23]. The variability in the study conclusions are possibly due to enrollment criteria and the definition of pre-keratoconus that was used by each study. Therefore, future validation studies are required to evaluate the predictive accuracy of IHD in these cases. 

**Index of Surface Variance**

The index of surface variance (ISV) reflects the deviation of the corneal radius with respect to the mean value [42]. High ISV values are observed in cases of irregular astigmatism. Simply, the ISV is an expression of the surface curvature irregularity. As demonstrated in Table 1, ISV is a highly sensitive parameter in distinguishing KC from normal eyes [52, 53, 57, 92]. Some studies have also indicated that ISV may be a superior index among the other tomographic parameters [57, 67]. 

Similar to IHD, there are promising studies that indicate ISV has a role in discerning pre-keratoconus [50, 52, 67]. Supporting data from these studies is shown in Table 2 and demonstrates the utility of ISV in screening patients for pre-keratoconus. Lastly, ISV may play a future role in management of the post-operative patient. In a study conducted by Kanellopoulous et al, SN for ISV was highest for tracking progression of KC [93], an important and valuable point to consider when monitoring patients longitudinally. 

**Index of Vertical Asymmetry**

The index of vertical asymmetry (IVA) characterizes the level of curvature symmetry data with respect to the horizontal meridian[42]. In distinguishing KC from normal eyes, IVA has been shown to have high SN and is capable of serving as a diagnostic parameter (Table 1) [52, 66]. While Hashemi and colleagues have also shown IVA to be a strong indicator in pre-keratoconus cases, this conclusion lacks reproducibility in other studies [52, 67]. Arbelaez et al studied an index similar to IVA that also showed high predictive power in detecting pre-keratoconus [94]. This was reaffirmed in other studies that showed IVA only second to BAD_D in predictive accuracy [50, 51].

Similar to Hashemi and colleagues, Bae et al showed that IVA was significantly different between normal and fellow eyes of individuals with unilateral KC. Importantly, they concluded that IVA was superior to BAD_D and ART (see below for discussion) in making this distinction [66]. As evidenced by our review, there is no consensus for the use of IVA in discrimination analysis of pre-keratoconus. While it is nonetheless a valuable index in distinguishing frank KC, we cannot conclude its validity of use for patients with suspected pre-keratoconus.

**Minimal Sagittal Curvature**

The radius of a sphere and its curvature are inversely proportional, thus the minimal or minimum radius of curvature (Rmin) is an index that corresponds to the point of maximum anterior curvature [90]. Similar to other topometric indices, Rmin has diagnostic efficiency in discriminating normal eyes from clinical KC as demonstrated in Table 1 [55]. Most studies that evaluated corneal tomography did not exclusively study Rmin as a predictive index. Though it has been validated by studies in discerning normal and clinical KC, our discussion on its practical use as a solo parameter is hindered by a lack of validation studies. In the study by Bae et al, Rmin failed to discern normal and fellow eyes of patients with KC [66]. Of note, Kanellopoulos and Asimellis identified Rmin as the index with the best correlation with best spectacle-corrected distance visual acuity, which may indicate a future role in monitoring post-LASIK outcomes and longitudinal visual function [93]. 

**Posterior Elevation**

Anterior and posterior elevation of the cornea can be mapped relative to a standardized reference shape such as a circle or ellipsoid and are standard displays of the Pentacam system. Posterior elevation (PE) represents the maximum PE in a zone above the standardized reference shape, which is typically a best fit sphere (BFS) or best fit torric ellipsoid (BFTE) [90, 91]. Belin introduced the enhanced BFS, which modifies the traditional BFS by excluding a 3.5 mm diameter area that is centered on the thinnest point of the corneal surface [26]. The advantage of the enhanced BFS is to avoid undue influence of the area surrounding the thinnest point of the cornea which otherwise causes a “steepening effect” due to pronounced protrusion [90]. By removing this “steepening” effect, the reference shape allows for enhancement of the ectatic area, which in turn improves recognition of ectatic changes and islands of elevation [26]. Among ophthalmologists, there is a growing consensus that PE data is the best diagnostic in identifying subtle keratoconic changes [50, 62, 63, 91, 95-98].

A review of the studies contributing to the rise of PE reveals its growing role in the clinical setting. In an elegant study comparing anterior and posterior corneal elevation data, Ishii and associates first found that elevation differences correlated well with the Amsler-Krumeich severity index [87]. Additionally, their study identified a larger AUC for PE differences when compared to anterior elevation differences. This data supported the hypothesis that PE changes occur first in the pathogenesis of KC. Since then, several studies have evaluated the diagnostic value of PE in distinguishing KC and pre-keratoconus [54, 65, 69, 87, 88, 99], which are summarized in Tables 1 and 2.

In terms of selecting a reference shape, the literature reports BFTE superior to BFS for screening, but that both reference shapes have limitations in identifying pre-keratoconus [88, 100, 101]. However, caution must be used in applying alone as an isolated measure. Nevertheless, there are also studies that demonstrate no statistical difference in PE when using different reference shapes [57, 63, 102]. Based on our review, we recommend the use of PE with either BFTE or BFS for screening of clinical KC. For cases of pre-keratoconus, however, the literature does not identify PE as a reliable individual parameter regardless of reference shape.

Detractors of PE argue that anterior curvature data may in fact have higher discriminative potential [66] and that PE abnormalities do not necessarily occur before anterior changes [72]. However, the multitude of studies above and experts in the field such as Saad and Gatinel have shown that PE data is often the earliest indicator of ectatic change [103]. The reason for different conclusions remains unclear, but study population and selection criteria certainly play a role in the determination of study outcome. In summary, the measurements of PE and PE differences are effective indices in aiding with the diagnosis of patients with KC, and their advantage over other measurements is significant.

**Pachymetric Indices**

The integration of pachymetry mapping can aid in determining severity of KC [42, 74, 104-106] and is indispensable in the evaluation and screening of refractive surgery candidates. A clinical reference summary of the best available pachymetric indices can be found in Tables 3 and 4. 

**ABCD Grading System**

One of the newest parameters, the ABCD classification, was introduced by Belin and Duncan as a staging system that incorporates tomographic and anatomical data and stratifies patients in a similar fashion to the existing Amsler-Krumeich classification [107]. Unlike the Amsler-Krumeich, this newest classification incorporates anterior (A) as well as posterior (B) radius of curvature, thinnest corneal pachymetry (C), and best corrected distance visual acuity (D) [107]. In addition, there is a modifier for scarring, categorized based on whether the scarring obscures iris details. 

While the ABCD classification system creates a unified system with valuable clinical utility, it has yet to be integrated in a widespread fashion likely in part due to lack of familiarity with radius of curvature as a tomographic parameter. Nevertheless, multiple studies have shown the value of PE data [63, 91, 98, 102, 108] and thus the ABCD system is a novel method for stratifying stages of KC. 

Given its recent introduction, the system requires further external validation but there are a few studies that have explored its application [109, 110]. In another interesting retrospective study by Imbornoni and colleagues, the ABCD classification system successfully identified five cases of true unilateral KC over a longitudinal period [111]. This points to the clinical value of using the ABCD system in early screening as it integrates posterior elevation data. It may also indicate that unilateral KC in the absence of environmental or mechanical factors is underreported as current methods of identification rely on anterior elevation maps only. 

**Pachymetric Progression Index: Average, Minimum, Maximum**

Pachymetric Progression Index (PPI) represents the change in corneal thickness and can be calculated over all 360 degrees of the cornea. The average of these meridians is represented as PPI-Avg, whereas the meridian with maximal pachymetric increase is PPI-Max, and minimal pachymetric increase is PPI-Min. Ambrósio reported the mean and standard deviation of the PPI-Avg, PPI-Max, and PPI-Min in a normal population to be 0.13 ± 0.33, 0.85 ± 0.18, and 0.58 ± 0.30, respectively [17]. If corneal thickness abruptly increases from the thinnest point towards the periphery, then expectedly the pachymetric index in that meridian will be higher [112]. Moreover, in their sentinel study, Ambrósio and colleagues showed that pachymetric progression indices are significantly better than single-point pachymetric measurements in the identification of KC [74].

Ectatic corneas have a rapid rate of pachymetric progression as compared to normal corneas [74]. As with other pachymetric indices, it is well-documented that PPI parameters can reliably distinguish KC as shown in Table 3 [70, 73, 86]. As for pre-keratoconus, there are many studies that validate the use of PPI parameters with good to excellent diagnostic accuracy as shown in Table 4 [64, 73, 86]. Nevertheless, there are still studies that detract from this significance and report unreliable diagnostic accuracy (AUC < 0.90) for PPI parameters [55, 66]. Therefore, despite its demonstrated value in several studies, there is still evidence of limitation for PPI that restricts our recommendation of use. While the majority of studies indicate a high predictive accuracy for PPI, there is not a universal consensus in the literature that allows for its widespread acceptance in detecting pre-keratoconus.

**Ambrósio Relational Thickness: Average, Minimum, and Maximum**

The Ambrósio Relational Thickness (ART) measurement is calculated as the ratio between the thinnest point and the PPI [74]. Among the pachymetric derived indices, the ART values, which includes ART-Average (ART-Avg), ART-Minimum (ART-Min), and ART-Maximum (ART-Max), provide validated accuracy in identifying ectasia [17]. This novel parameter allows for differentiation of keratoconic corneas with relatively normal central corneal thickness [17, 74, 112].

While first introducing the novel parameter, Ambrósio et al recommended a 339 micrometer (µm) threshold for ART-Max, which had an AUC of 0.983 with 100% SN and 95.6% SP [1]. In the same study, ART-Avg, with a threshold of 424 µm had a mildly better AUC of 0.987 with 95.5% SN and 96.5% SP, but there was no significant difference between ART-Max and ART-Avg in discerning normal and keratoconic eyes [1]. Wahba et al evaluated the accuracy of pachymetric indices using different reference shapes in a recent study and concluded that the ART-Max tied for the highest AUC (0.987) at a cut-off value of 412 µm [73]. These results are comparable to the original study by Ambrósio et al that introduced the relational thickness parameters [74].

Subsequent studies have successfully validated ART parameters as diagnostic indices for distinguishing KC (Table 3) [57, 70, 85]. For pre-keratoconus, there are inconsistencies in the literature regarding the diagnostic accuracy of ART indices (Table 4) [66, 69, 72]. These differences may be in part due to variability of study population or various selection criteria used to define pre-keratoconus. As a result of these inconsistencies, we recommend the use of ART indices for determining the presence of KC only. When using a cut-off value between 300 µm and 400 µm there is high predictive accuracy [106].

**Belin-Ambrósio Enhanced Ectasia Display Total Deviation Value**

Belin-Ambrósio Enhanced Ectasia Display Total Deviation Value (BAD_D) is a multivariate index that essentially gives the clinician a comprehensive global view of the cornea and helps to objectively screen patients for mild disease like pre-keratoconus [90]. Through a combination of pachymetric and curvature data, the BAD_D considers 9 separate indices that are summarized in a final ‘D’ value. This value is calculated based on regression analysis of the following indices: Df (deviation of the normality of the front elevation), Db (deviation of normality of the back elevation), Dt (deviation of normality of corneal thinnest point), Da (deviation of normality of Ambrósio relational thickness), Dp (deviation of normality in average pachymetric progression), Dy (displacement of thinnest point along the vertical meridian), anterior elevation at the thinnest point, posterior elevation at the thinnest point, and Kmax (Table 7) [26]. In the Pentacam display system, each parameter is indicated in yellow (suspicious) if it is ≥ 1.6 SD from the mean or in red (abnormal) if it is ≥ 2.6 SD from the mean. The final D value is based on a regression analysis and maximizes accuracy in the detection of ectasia.

In the first independent validation of BAD_D, the refractive index eliminated 99% of KC corneas and achieved a false positive rate of 0% when a cut-off of 2.69 was used [113]. Since then, BAD_D has been shown in multiple studies to have the highest accuracy in detecting both clinical KC and pre-keratoconus [14, 17, 51, 53, 67, 70, 72, 85]. Hashemi et al validated the diagnostic validity of BAD_D in a relatively large sample size of patients (n = 326). The study concluded that BAD_D is one of the best available Pentacam indices in identifying both definitive and pre-keratoconus [67]. In a comparison of corneal dynamic responses and tomographic measurements, Chan et al showed that BAD_D had the highest AUC (0.994) of any Pentacam parameter in discriminating KC from normal corneas [51].

Interestingly, Bae and colleagues did not find a significant difference in BAD_D between normal and fellow eyes in individuals with unilateral KC [66]. Perhaps this is attributable by the fact that analysis of fellow eyes was restricted to those with normal Pentacam indices, including elevation and pachymetric maps. This may allude to a potential limitation of BAD_D in detecting subtle morphologic change at baseline, and perhaps points towards its value as a progressive index to be followed over time.

An interesting retrospective study that compared preoperative parameters of patients with post-LASIK ectasia and those with stable outcomes also identified BAD_D as the most accurate parameter in identifying preoperative risk [114]. By combining susceptibility parameters and procedure-related parameters such as percent tissue altered (PTA) and residual stromal bed (RSB) there may be improved risk stratification of patients. In the future, it is likely that BAD_D will be incorporated with biomechanical properties to yield a better diagnostic test. Some studies have already begun to explore this use of BAD_D [75], and the results are promising.

**Central Corneal Thickness**

Central corneal thickness (CCT) is a fundamental pachymetric index that is the basis of identifying corneal thinning disorders [5] and has been a mainstay screening index for nearly thirty years, especially in the presence of topographic asymmetry [115-118]. While CCT has been validated to differentiate between normal and KC eyes, it has largely been replaced with indices that carry much higher sensitivities. As early as 2003, Ambrósio and colleagues began exploring the topographic characteristics of poor candidates for refractive surgery and identified the importance of corneal thickness in risk stratification [115-118].

Ambrósio and colleagues were the first to introduce the corneal-thickness spatial profile, which found a statistically significant difference in corneal thickness between normal eyes and those with KC [119]. Though their study analyzed corneal thickness beyond a central point, it also highlighted the value of CCT as a tool in the preoperative screening process. Subsequent studies evaluating CCT have confirmed its diagnostic utility as a screening index for KC [80, 112]. Conversely, there are disagreements in the literature for the use CCT in pre-keratoconus. While some studies validate its use as a diagnostic parameter, other studies have failed to find significant differences (Table 4) [66, 77, 86, 103]. As with other indices that have this same disparity, we believe the varied selection criteria used for pre-keratoconus influences the outcome analysis.

In conclusion, CCT as a single measurement has documented limitations for long-term follow up and detecting pre-keratoconus [120, 121]. While initially CCT was considered a valuable index for KC, it has largely been modified to include the vast amount of additional information provided by tomographic devices. Despite its shortcomings, CCT is still a valuable parameter in identifying KC; however, it should not be relied on exclusively to exclude or diagnose ectasia.

**Pentacam Random Forest Index**

The Pentacam random forest index (PRFI) was first introduced by Lopes and colleagues [58]. The origin of its name comes from the random forest artificial intelligence model, which was generated using Pentacam parameters. In this novel study, PRFI had an AUC of 0.992 (99.4% SN, 98.8% SP), which was statistically superior than the BAD_D when assessing all ectasia cases [58]. As a result, the study investigators concluded that PRFI enhanced ectasia diagnosis. Their study highlights the implications of machine-learning algorithms in corneal tomography. As there are a wide number of refractive indices to evaluate, perhaps in the future computational analysis will be better handled by a machine-learning program rather than a subjective operator.

Despite the promising initial results of the PRFI, it still misclassified up to 20% of cases with pre-keratoconus. In fact, in another recent study, the third-generation BAD_D outperformed the PRFI [59]. Future research should integrate topometric, pachymetric, and biomechanical parameters to develop a better understanding of the corneal surface and identify the earliest changes in structure.

**Thinnest Corneal Thickness**

In addition to the indices mentioned above, the pachymetric map identifies the thinnest corneal thickness (TCT) as part of the detailed distribution map [90]. TCT is a valuable diagnostic parameter in detecting primary ectatic disease [105]. Several studies have validated the use of TCT in the identification of KC as demonstrated in Table 3 [50, 54, 65, 66, 76, 92]. When compared to other Pentacam indices the AUC is generally lower; though we are unable to assess the statistical significance of these differences, it should be considered when evaluating the predictive accuracy of TCT when compared to other parameters. In a recent study by Xu et al, the application of the Zernike polynomial fitting method showed that TCT could discriminate between normal and pre-keratoconus eyes [60]. This postulates that Zernike polynomial modeling may have a role in improving diagnostic SN of refractive indices beyond the traditional scope of wave front fitting. This was corroborated by subsequent studies that show TCT is a superior parameter in distinguishing the various stages of KC [70, 77]. While the clinical utility of TCT continues to be explored, the available literature has confirmed its use for detection of KC. Though TCT may serve as a reliable parameter for detection of pre-keratoconus in the future, we do not recommend its use at this time for these cases.

**Other Scheimpflug Systems**

While this review article focuses on the Pentacam camera, there are several other Scheimpflug systems available on the market such as Galilei (Ziemer Ophthalmic Systems AG, Port, Switzerland), TMS-5 (TMS-5 (Tomey Corp., Nagoya, Japan), and Sirius (Construzione Strumenti Oftalmici, Florence, Italy). The Galilei system recently joined the arena of Scheimpflug imaging and offers the exciting addition of a dual-channel camera system. Given the additional KC predictive indices available on the Galilei system, a full discussion and evaluation of this camera can be found in the article, “Galilei Corneal Tomography for Screening of Refractive Surgery Candidates: A Review of the Literature, Part II”, which the second article in the three part-series put forth by the authors.

**Biomechanical Indices**

Biomechanical data is synergistically integrated with Scheimpflug parameters employed by the Pentacam system and improves identification of early ectasia. Corneal biomechanical parameters, such as fragility and weakness, are known to influence the susceptibility of developing ectatic disease [14, 122-125]. A variety of properties measured by both the Ocular Response Analyzer (ORA, Reichert, Buffalo, NY) and Corvis ST (Oculus Optikgeraete GmbH; Wetzlar, Germany) have promise in early ectasia detection. In combination with Pentacam HR, corneal biomechanics have the potential to modify refractive screening. Given that these parameters are independent of the Pentacam system, a full discussion regarding their impact and importance can be found in the article, “Advances in Biomechanical Parameters for Screening of Refractive Surgery Candidates: A Review of the Literature, Part III”, which is the final article in a three-part series put forth by the authors. 

## DISCUSSION

**Application and Interpretation of Pentacam Indices**

The clear majority of Pentacam indices are capable of discerning KC from normal eyes. However, it is frequently the identification of pre-keratoconus cases that poses a problem for ophthalmologists particularly when assessing pre-operative risk of ectasia. As evidenced by our review, the clinician should never solely rely on an index in the clinical decision-making process. Rather, by combining the relevant clinical data and patient demographics with the Pentacam indices, the clinician can navigate risk stratification in a deductive fashion. It is well known that certain geographic areas have a higher incidence of KC [126-130]. Potentially in these regions there should be a lower index of suspicion when deciding which patients are suitable candidates for corneal refractive surgery and the ophthalmologist should always err on the side of caution in borderline cases.

Based on our review, the single best index available on the Pentacam system currently is BAD_D, with secondary consideration for ART and PPI values. Our recommended optimized cut-off values for each index are highlighted in Table 6. Ultimately, the crucial recommendations are for identification and screening of pre-keratoconus cases. This recommendation is based on our comprehensive review and should not be used alone to guide clinical decision-making. Rather, we encourage to employ simplified recommendations in Table 6 as a quick screening tool when there is already a high index of suspicion, especially in cases of pre-keratoconus. Most importantly, the cut-off values are not intended for use as individual diagnostic parameters. The utility of our recommended values is in the context of side-by-side comparison; for example, if an individual meets pre-keratoconus criteria for multiple refractive indices, then it should be considered a red flag. Many of the indices discussed in this review can discriminate normal and KC. However, in pre-keratoconus cases, our recommendation is to combine multiple indices, namely BAD_D, ART, and PPI, along with clinical judgment in order to successfully risk-stratify each patient. Interestingly, all three of these parameters are categorized as pachymetric indices. Whether this is simply based on presently available literature is unclear, however, future studies should aim to compare pachymetry and tomography measurements to identify if one is superior to the other.

**Figure 8:**
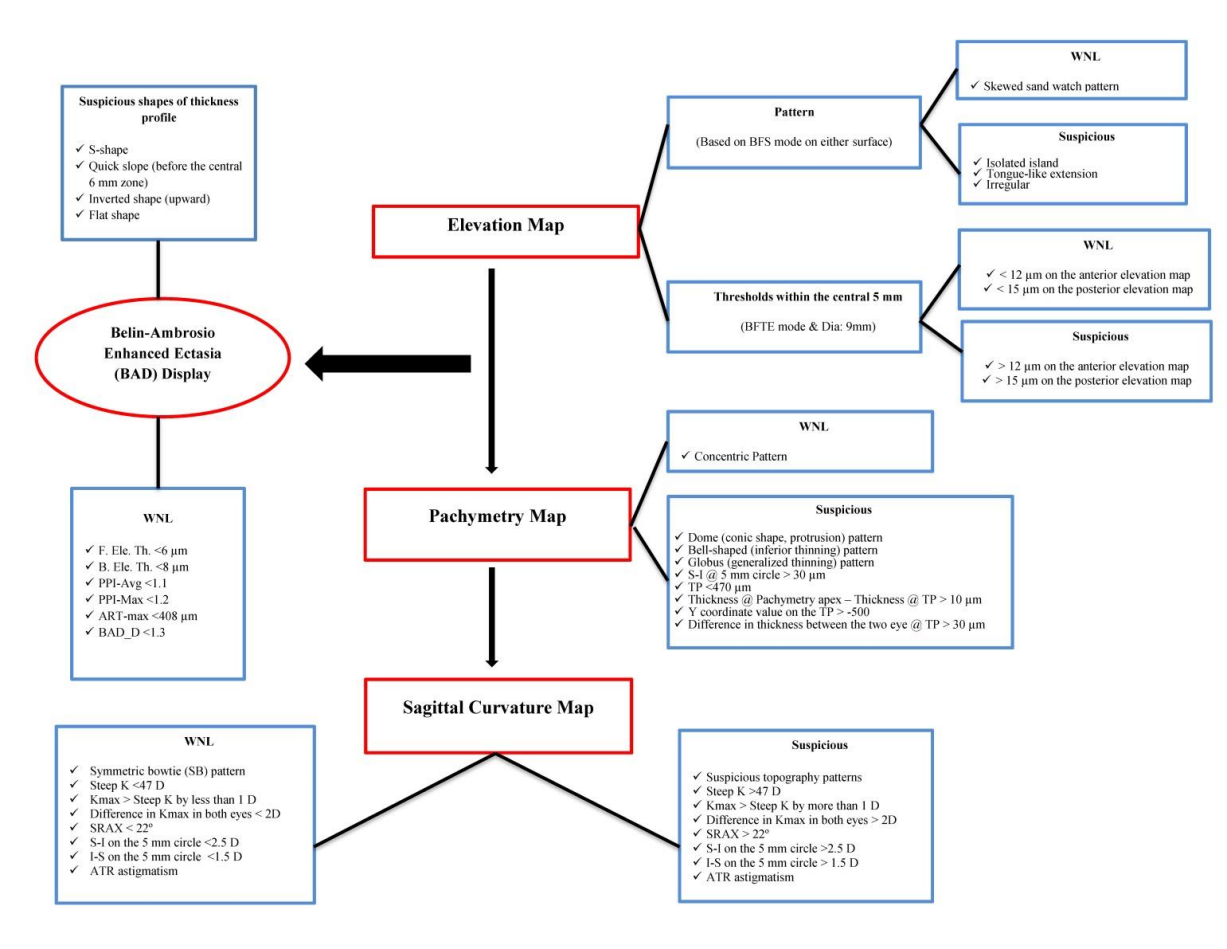
The Pentacam Road Map. A step-wise algorithm that can assist in screening surgical refractive candidates

Abbreviations: ART-Max: maximum Ambrósio relational thickness; ATR: against the rule; BAD_D: Belin-Ambrósio enhanced ectasia display total deviation value B. Ele. Th.: back elevation thickness; BFS: best-fit sphere; BFTE: best-fit toric ellipsoid; D: diopter; Dia: diameter; F. Ele. Th.: front elevation thickness; I-S: inferior-superior; K: keratometry value; Kmax: max keratometry value; PPI-Max: maximum pachymetric progression index; PPI-Avg: average pachymetric progression index; S-I: superior-inferior; SRAX: skewing of the steepest radial axis; TP: thinnest point; WNL: within normal limits.

We recommend the use of elevation, pachymetry, and sagittal curvature maps in a step-wise approach to evaluating a surgical candidate. As demonstrated in Fig. 8, there are particular patterns, shapes, and thresholds to be mindful of when evaluating the corneal surface with Pentacam. In addition to the values provided in Table 6, we hope this can serve as a useful road map when screening patients in clinic.

An additional point to consider is interpretation of seemingly normal indices when comparing a set of eyes. Though an individual may have tomography within normal ranges, a large disparity between eyes should raise concerns [131]. Despite the advancement of indices, analysis should always be bilateral and include a thorough evaluation of patient’s past medical and family history with close attention in particular to family members who have previously had refractive surgery.

**Limitations**

Establishing a comprehensive normative database has been the subject of several studies [132-137]. This is valuable as normal limits of variance are fundamental to understanding the spectrum of ectasia. Beyond this, there are expected changes in the myopic and hyperopic corneas that also contribute to normal geographic variation of the cornea. While many normal values are defined by the Pentacam system, it is also imperative to recognize that non-modifiable factors such as race, age, and gender may also influence the normative range.

As pointed out in the study by Guber et al, another limitation to consider when detecting KC is that measurement error is significantly greater in ectatic than healthy eyes [28]. Another limitation of Scheimpflug imaging is that it relies on fixation of the eye, which is more difficult in keratoconic eyes compared to normal subjects. A recent study shows that there are significant differences between Pentacam and other systems in all curvature measurements [138], which highlights the importance of developing a normative database with respect to Pentacam alone.

Instrumentation aside, the greatest limitation confronting appropriate refractive screening is the lack of agreement in defining pre-keratoconus. The variability in conclusions from study to study is most likely attributable to the different definitions used for subclinical KC, suspect KC, and FFKC, all referred to in this paper as pre-keratoconus. Refractive surgeons may never agree on a consensus definition, but our recommendation for the future is to adjust index cut-off values based on the population that is being analyzed and to report this accordingly. In the current literature, investigators are reporting only optimized cut-off values, but from a database standpoint it would be advantageous to publish whole datasets instead of optimal values only.

Pentacam indices have not been cross-validated for the various reference surfaces. While BFS is the most commonly used and intuitive reference shape, it may not be the reference shape of choice in patients with atypical changes including irregular astigmatism or cases with high corneal astigmatism [139]. The BFS is the best reference for identifying the location of a cone, but the BFTE is the best reference to identify the height of the cone. As discussed, the enhanced BFS may be superior to both the conventional BFS and BFTE [26]. Thus, future studies are required to investigate the repeatability of refractive indices with each reference shape. Ultimately, if a uniform understanding of pre-keratoconus (subclinical ectasia) is defined, then it will simplify the task of effectively comparing indices and setting universal cut-off values for the Pentacam system. Until then, however, identifying outliers and poor candidates for refractive surgery will remain a challenging clinical task. 

**Looking Ahead**

Application of machine learning algorithms and neural networks has been explored to assist in the identification of corneas with pre-keratoconus and KC [56, 140, 141]. Kovács and colleagues trained machine learning on bilateral data in individuals with unilateral KC [56] and then sought to evaluate diagnostic accuracy of their classifiers. This study found that machine learning was superior in discriminating eyes with pre-keratoconus compared to the best unilateral single parameter. As far as discriminating eyes with clinical KC, however, there was no significant improvement with machine learning diagnostic accuracy. This may indicate a niche for the application of highly sensitive and specific neural networks in identifying pre-keratoconus, as clinically these are the most challenging to identify. Nevertheless, a limitation of this study was exclusion of certain parameters, notably BAD_D, which is of primary relevance in KC detection as discussed above. Future studies should include all parameters when developing training sets for machine learning classifiers.

Another consideration for the future is identifying the refractive indices best suited to monitor for progression of KC as this can influence treatment protocols and intervention. A recent study by Martinez-Abad et al established a progression index that could predict the level of KC progression in non-surgically treated patients [142]. However, with a small sample size, the reference model is limited in its predictive value. Future studies with longer follow-up are needed to assess the accuracy in predicting progressive disease. Nevertheless, defining a parameter for progression is critical in navigating treatment protocol for patients.

Outside of the indices that assess pre-operative risk, there are many factors that influence the chance of developing ectasia after surgery. These include RSB, thin cornea, age, chronic trauma, persistent eye rubbing, and high myopia [131, 143]. Age is often overlooked, but is likely the most important defining characteristic an individual’s intrinsic biomechanical properties. In one study, PE, PPI-Avg, PPI-Max, ART-Avg, and ART-Max were all found to have significant differences among three age-categorized groups [144]. This further emphasizes the importance of considering demographics and risk factors that influence ectasia development beyond those measured by the Pentacam system.

Another direction for future research is determining factors that are associated with post-surgical ectasia including obesity, obstructive sleep apnea (OSA), gender, and genetics. Several studies have also shown that obesity may play a role in the etiology of KC [145-148], though the exact pathophysiology remains unclear. OSA has also been independently investigated regarding its role in KC [147, 149-151]. Another important point to consider is that both OSA and obesity are conditions associated with floppy eyelid syndrome [146, 147, 150, 152-154]. We speculate, thus, that floppy eyelid syndrome may pose as a confounder in patients with KC. It is also possible that obesity and OSA contribute to tarsal laxity, which in effect makes the cornea more vulnerable to mechanical irritation, a known risk factor for KC [143, 146, 155-158].

Gender also remains a factor to consider as several studies have demonstrated a younger age of onset in male patients [129, 159-163]. However, there is a possibility of multiple confounders and the potential for effect modification in these studies such as race, ethnicity, and age-related differences. Lastly, there is evidence to suggest that there is a genetic component to KC [164-175]. Still, however, there is an incomplete understanding of the genetic component and how it is influenced by environmental susceptibilities such as chronic eye rubbing, sun exposure, geographic location, and atopy [127]. Subsequent investigations should also consider these intrinsic risk factors and potentially their integration with individual patient adjustment of the cutoffs for indices measured by the Pentacam device.

## CONCLUSION

Corneal tomography and Scheimpflug technology have advanced screening of the refractive surgery candidate. As evidenced by our review, the many refractive indices on the Pentacam system are reliable parameters for identification of KC. One large obstacle confronting modern day tomography is the lack of unified nomenclature and classification criteria of preclinical ectasia (referred to in this paper pre-keratoconus), which translates to difficulty in detection of this enigmatic entity. Inconsistencies in the literature when evaluating refractive indices in patients with pre-keratoconus exacerbate the issue. For each study that successfully identifies a parameter for these cases, there seems to be a detracting study that counters the index. Thus, we encourage future studies to be transparent in their selection criteria and population data in order to adequately compare investigations. Nevertheless, the literature confirms that corneal tomography can be used reliably in differentiating healthy eyes from eyes with KC and offers great promise in the evaluation of pre-keratoconus. Going forward in the evaluation of pre-keratoconus, the best approach is a combination of refractive indices as detailed in our recommendations above aided with a comprehensive evaluation of the patient’s clinical picture.

## DISCLOSURE

Ethical issues have been completely observed by the authors. All named authors meet the International Committee of Medical Journal Editors (ICMJE) criteria for authorship of this manuscript, take responsibility for the integrity of the work as a whole, and have given final approval for the version to be published. No conflict of interest has been presented.

## Funding/Support:

Research to Prevent Blindness, NY, USA
